# NIPTmer: rapid k-mer-based software package for detection of fetal aneuploidies

**DOI:** 10.1038/s41598-018-23589-8

**Published:** 2018-04-04

**Authors:** Martin Sauk, Olga Žilina, Ants Kurg, Eva-Liina Ustav, Maire Peters, Priit Paluoja, Anne Mari Roost, Hindrek Teder, Priit Palta, Nathalie Brison, Joris R. Vermeesch, Kaarel Krjutškov, Andres Salumets, Lauris Kaplinski

**Affiliations:** 1 Institute of Molecular and Cell Biology, University of Tartu, Tartu, Estonia; 2 Department of Obstetrics and Gynaecology, Institute of Clinical Medicine, University of Tartu, Tartu, Estonia; 3 Women’s Clinic, Tartu University Hospital, Tartu, Estonia; 4 Competence Centre on Health Technologies, Tartu, Estonia; 5 Department of Biomedicine, Institute of Bio- and Translational Medicine, University of Tartu, Tartu, Estonia; 6 Estonian Genome Center, University of Tartu, Tartu, Estonia; 7 Center for Human Genetics, KU Leuven, Leuven, Belgium; 8 Department of Biosciences and Nutrition, Karolinska Institutet, Huddinge, Sweden; 9 Molecular Neurology Research Program, University of Helsinki and Folkhälsan Institute of Genetics, Helsinki, Finland; 10 Department of Obstetrics and Gynecology, University of Helsinki and Helsinki University Hospital, Helsinki, Finland

## Abstract

Non-invasive prenatal testing (NIPT) is a recent and rapidly evolving method for detecting genetic lesions, such as aneuploidies, of a fetus. However, there is a need for faster and cheaper laboratory and analysis methods to make NIPT more widely accessible. We have developed a novel software package for detection of fetal aneuploidies from next-generation low-coverage whole genome sequencing data. Our tool – NIPTmer – is based on counting pre-defined per-chromosome sets of unique k-mers from raw sequencing data, and applying linear regression model on the counts. Additionally, the filtering process used for k-mer list creation allows one to take into account the genetic variance in a specific sample, thus reducing the source of uncertainty. The processing time of one sample is less than 10 CPU-minutes on a high-end workstation. NIPTmer was validated on a cohort of 583 NIPT samples and it correctly predicted 37 non-mosaic fetal aneuploidies. NIPTmer has the potential to reduce significantly the time and complexity of NIPT post-sequencing analysis compared to mapping-based methods. For non-commercial users the software package is freely available at http://bioinfo.ut.ee/NIPTMer/.

## Introduction

Non-invasive prenatal testing (NIPT) is an expanding clinical application in which circulating cell-free DNA (cfDNA) in the pregnant woman’s blood is analyzed, usually by next-generation sequencing (NGS), to detect potential fetal chromosomal aneuploidies. This approach is possible since a sizable fraction (3–22%) of cfDNA in the maternal blood has a fetal origin^1,2^. Several applications detect chromosomal aneuploidies with high sensitivity and specificity by calculating the relative coverage of all chromosomes in sequenced reads and comparing these numbers against a set of reference samples using standard statistical operations^3,4^. Most of these include a mapping stage – reads aligning to the reference genome, which is computationally demanding and requires expertise to produce uniform results across samples and populations. Therefore, innovative data analytical solutions are needed to simplify and improve NGS data analysis, like in NIPT, to make it more accessible in clinical environment.

Here, we introduce a k-mer-based (k-mer is a short nucleotide sequence with fixed length k) NIPT analysis method, referred to as the NIPTmer, a novel software package and workflow process in which the mapping of reads is replaced with counting a predefined set of k-mers straight from the FASTQ formatted sequencing raw data. Using this approach, NGS data can be analyzed on high-end desktop workstation or a low-end server, 1 to 2 orders of magnitude faster compared to mapping-based methods.

## Results

### Chromosome-specific k-mer lists

Our methodology and its implementation relies on counting pre-defined per-chromosome sets of unique k-mers from the raw sequencing data, applying a linear regression model to the chromosome-specific k-mer counts of each studied sample and comparing the predicted and observed k-mer counts.

The k-mer length of 25 bases was chosen for this study (see Discussion). By filtering, we excluded (i) k-mers that have multiple copies in genome, (ii) k-mers that overlap with common polymorphisms and (iii) k-mers that overlap with low-complexity regions (such as telomeres and centromeres) and pseudoautosomal regions of chromosomes X and Y. In addition, we removed those k-mers that were specific in human reference genome, but for unknown technical or biological reasons were over- or underrepresented in the sequenced healthy euploid individuals (see Methods).

**Figure 1:**
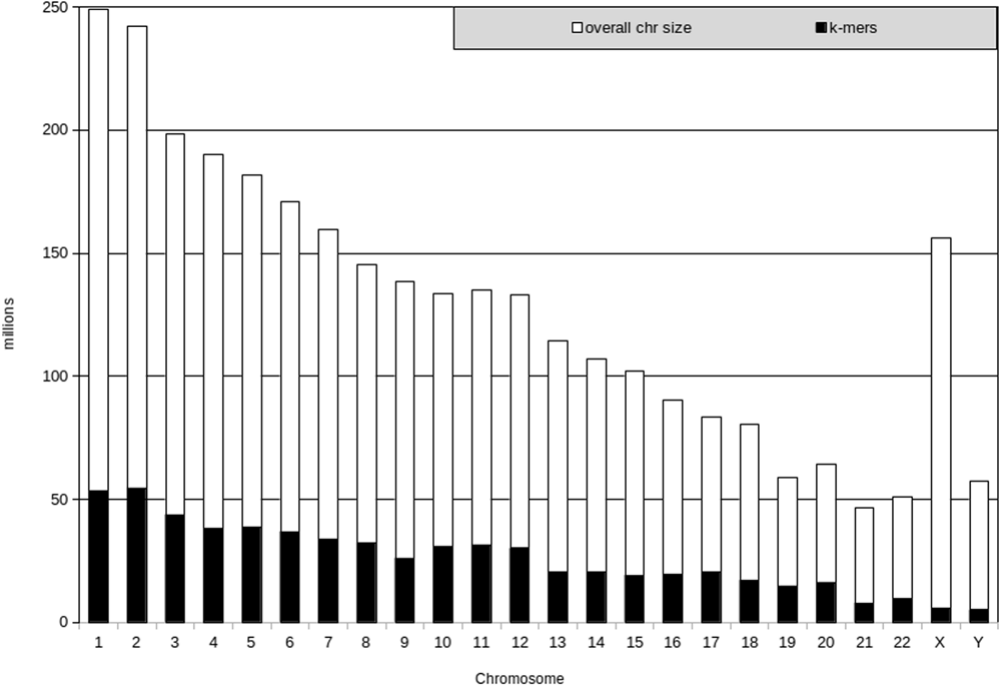
The number of 25-mers in the final k-mer lists used in the NIPT analysis (black) and the maximum number of possible k-mers (white) for each chromosome. The final k-mer lists for autosomes ranged from 7,781,826 (chr 21) to 54,629,103 (chr 2) unique 25-mers. The lists for chromosomes X and Y were the smallest: 5,860,559 and 5,071,089 unique 25-mers, respectively.

The final k-mer list sizes varied between 54,629,103 (chromosome 2) and 5,071,089 (chromosome Y). This constitutes about 21.5% (16.7–25.4%) of all possible k-mers in autosomes (Fig. 1). The usable k-mers in chromosomes X and Y represented a considerably smaller fraction of all possible k-mers.

K-mer list creation and filtering is the most resource consuming process. However, it has to be done only once per platform and per population, and once created, the same lists are used for all subsequent analyses. K-mer lists generated for testing our application are available from the NIPTmer download site.

### Counting k-mers from reference samples and constructing data-matrix

We constructed 25-mer lists from all sequenced reference samples and intersected it with chromosome-specific k-mer lists, obtaining the number of chromosome-specific k-mers present in a particular sample. The ratio of the chromosome-specific k-mers detected in the sample to the total number of k-mers in corresponding chromosome-specific k-mer list gives the relative sequencing coverage of a given chromosome.

The resulting data was a 25 × 583 matrix, where the rows represented different samples and the columns – 22 autosomes, chromosomes X and Y, and overall GC content in the sample.

### Aneuploidy detection

Due to unknown biological and technical variance, the detected per-chromosome coverage differs from the expected value when only the total sequencing depth is taken into account. This variance is partially predictable by a linear regression model that uses the detected coverage of other chromosomes as independent variables. Although, the individual coverage values of chromosomes vary between samples, they have a certain global pattern that can be used to predict the expected coverages with higher precision. The difference between the observed and predicted coverage is further normalized by the variance in reference samples. The standardized relative difference (z-score) between predicted and observed values is expected to be normally distributed, so by setting the cut-off at 3.5 standard deviations, we should get a false positive rate at <0.05%.

### NIPTmer testing and validation

To estimate the accuracy of our method and the NIPTmer software package, 294 cfDNA samples collected at Tartu University Hospital (Tartu, Estonia) were used. The cohort included five samples from pregnancies with trisomy 21 (T21), four samples with trisomy 18 (T18), and one mosaic T18 sample. The minimum sequencing coverage of samples was 0.08, average 0.32 and maximum 0.42.

**Figure 2:**
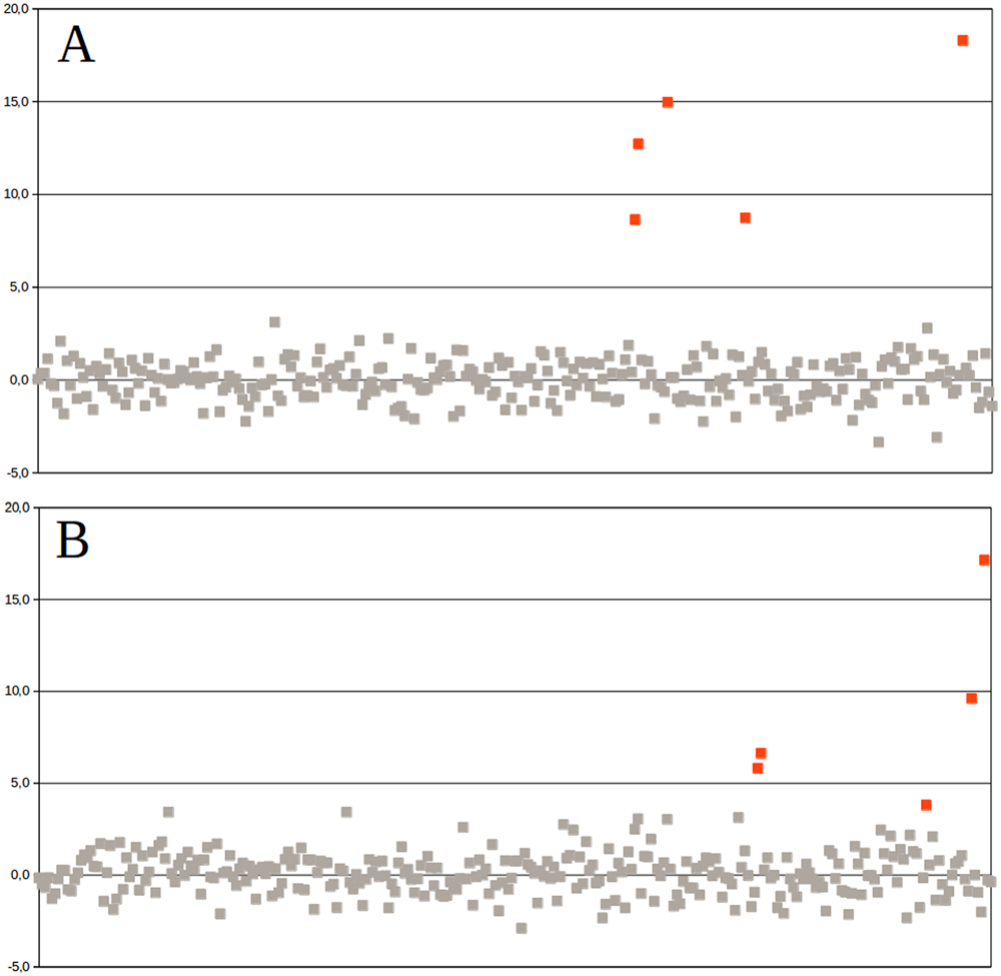
NIPTmer results for 294 subjects of Estonian cohort collected from Tartu University Hospital. Chromosome 21 (**A**) and chromosome 18 (**B**). Samples are ordered on the x-axis by the order of subject enrollment. The y-axis represents the standardized deviation (z-score) between the predicted and observed coverage of a given chromosome. Euploid samples are represented by gray points and aneuploid samples by red points. If we apply the cut-off line at 3.5 SD, all five T21, four T18 samples and one mosaic T18 case (z-score = 3.8) are recognized.

The NIPTmer algorithm discriminated between normal and trisomy cases for all T21 and all non-mosaic T18 pregnancies with the z-score being more than 5 standard deviations (SD) greater than normal samples (Fig. 2). There was only a slight difference between z-scores of the mosaic T18 and the highest normal sample. The data-matrix and z-score matrix derived from our sample set are available in the supplementary materials (Supplementary files 1 and 3). The sex of the fetus was determined correctly for all samples.

**Figure 3:**
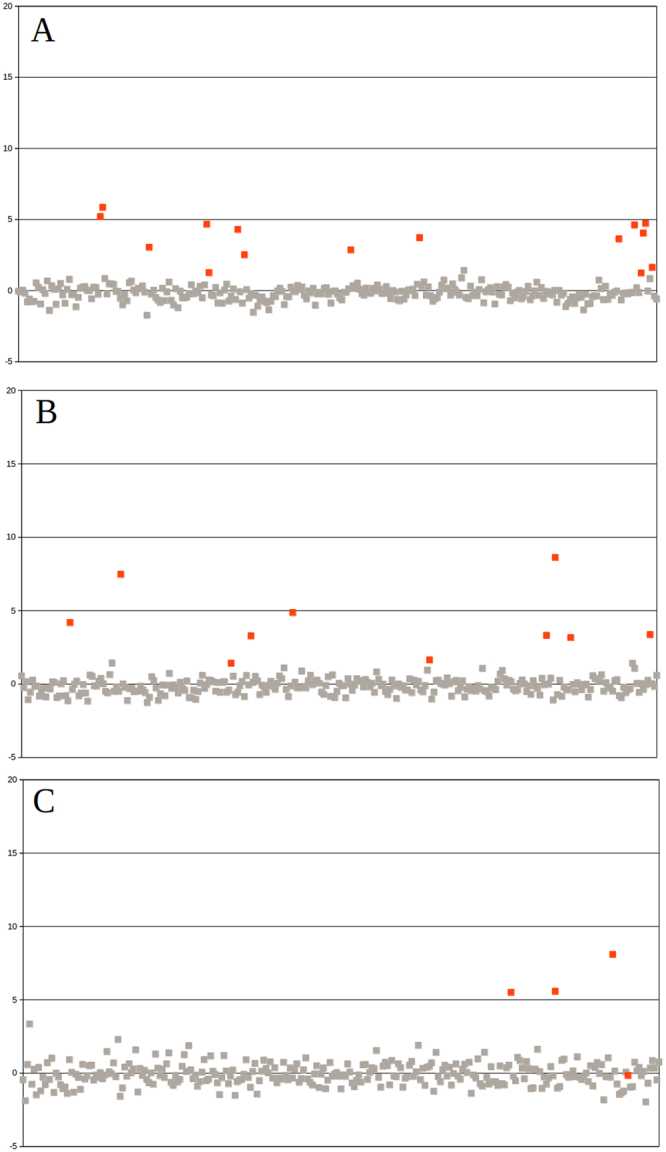
NIPTmer results for 289 subjects of Belgian cohort. Chromosome 21 (**A**), chromosome 18 (**B**) and chromosome 13 (**C**). Samples are ordered on the x-axis by the order of subject enrollment. The y-axis represents the standardized deviation (z-score) between the predicted and observed coverage of given chromosome. Euploid samples are represented by gray points and aneuploidy samples by red points. All non-mosaic aneuploidies, including T21 (15/15), T18 (10/10), and T13 (3/3), had higher z-scores than the highest control score, although the difference between lowest trisomy and highest normal score was small for trisomies 18 and 21. Unfortunately, we could not detect one mosaic T13 with our pipeline.

In addition, NIPTmer was blindly validated on sequencing data of 289 cfDNA samples of a validation cohort from Belgium, which had been described elsewhere^3^. All non-mosaic aneuploidies, including T21 (15/15), T18 (10/10), and trisomy 13 (3/3), had higher z-scores than the highest control score, although the difference between lowest trisomy and highest normal score was small for trisomies 18 and 21. A single mosaic T13 remained undetected (Fig. 3). The data-matrix and z-score matrix derived from the validation set are available in the supplementary materials (Supplementary files 2 and 4). The lower discriminative power of our model on validation cohort can be explained by larger stochastic variance in validation cohort, caused by lower sequencing coverage. In Estonian cohort the average coverage was 0.32, in Belgian cohort 0.13.

### NIPTmer computational performance

Software performance tests were conducted on a 32-core CentOS 5.10 Linux server (2.27 GHz and 512 gibabytes of RAM). The first step (list preparation) is the most time-consuming and, depending on the number of control individuals, takes one to several days. The second step, creation of the k-mer list from the sequencing samples and counting unique k-mers takes 4 to 5 minutes with 180% CPU load. On a laptop with Intel Core i7 processor and 8 GB of RAM, the creation of lists and counting unique k-mers took 20 to 25 minutes while using an external drive, and about 10 minutes when data was on the internal SSD. The third step, model building and z-score calculation took less than 1 second for 294 samples on either platform.

## Discussion

NIPTmer is an innovative tool for the rapid and accurate detection of fetal aneuploidies from NGS reads. It works by directly counting validated k-mers from sequencing reads and avoids time- and resource-consuming mapping of sequencing reads to reference genome, which is a part of most NGS data analysis pipelines^5–8^.

One of the most crucial and time-consuming parts of the majority of NIPT pipelines is calculating the relative chromosome coverages from raw sequencing data. The most commonly used method is to map the reads to the reference genome and to estimate the coverage by counting the number of reads mapped to each chromosome^5–8^. Due to known issues in mapping certain genomic regions, this step also involves removing reads that were mapped to known areas that tend to skew the sequencing results. In NIPTmer, we first construct k-mer lists by identifying the sequences (i.e. 25-mers) that represent a particular chromosome the most consistently in reference data, and then count only these k-mers directly from the raw sequencing reads. This results in saving of both time, as mapping is computationally demanding, and storage space, as there is no need to keep intermediary full mapping files, of which only small part is used. The mentioned advantages allow, for example, the constant updating of the reference group making the analysis more precise, which is critical in terms of clinical application. In addition, reads mapping and relative chromosome coverages calculation operations require higher expertise compared to the straightforward k-mers counting, and thus the use of NIPTmer in clinical environment would probably be more convenient.

The discriminative power of the NIPTmer method depends both on the quality of DNA preparation and sequencing, and per-chromosome k-mer lists. Good k-mer lists should represent each chromosome uniformly and uniquely. We used the k-mer length of 25 bases, which is a compromise between specificity and sensitivity^9^. Shorter k-mers are less specific and would thus result in shorter k-mer lists and higher stochastic variance of total k-mer counts, making the detection of true variance more difficult. Longer k-mers, although more specific, have slightly higher probability of remaining undetected due to sequencing errors or novel mutations that are difficult to consider when the k-mer lists are prepared. We analyzed the distribution of final unique 25-mers across each chromosome and did not find any clear bias, except the expected lack of unique k-mers at the long low-complexity regions like centromeres and telomeres (data not shown). Therefore, in our opinion, increasing k would not have contributed to representing the chromosomes more evenly.

We applied multi-step filtering to preliminary k-mer lists, to obtain a set of k-mers that have low variability of coverage in most samples and are, thus, suitable for the estimation of relative amount of DNA. It is also possible that k-mer lists can be further improved by optimizing the whole selection procedure to minimize the variability of total k-mer counts in normal individuals by choosing the optimal combination of included polymorphisms, the number of population controls and cut-off values. As this would require substantially larger control dataset, preferably including individuals from different populations, it remains for future work.

In NIPTmer, samples are further analysed by standardizing (z-scoring) the difference of the per-chromosome coverage and the predicted coverage for the same chromosome. The z-score is used as an indicator of aneuploidy risk for a certain chromosome. However, even most conservative k-mer lists do not guarantee a uniform coverage across euploid chromosomes over all samples. The relative coverage (compared to the mean of the whole genome) of different chromosomes varies both between chromosomes and between samples. We found that there was a certain pattern in the relative coverages of chromosomes, i.e. certain groups of chromosomes showed a tendency to have either smaller or higher coverages in a sample. We also found that the average GC content of sequenced reads had strong correlation with the relative coverage of different chromosomes. In addition, the average length of library fragments correlated with per-chromosome coverages, but due to unreliable measurements, we could not use this observation in our calculations. As the relative coverage of different chromosomes had certain pattern, we assumed that it could be estimated by a linear model. The model predicts expected coverage of the chromosome of interest by using the coverages of all other chromosomes and GC content as independent variables. The procedure also allows including additional variables that may describe additional relevant parameters of the sample. The more detailed description of the relation between the coverages of different chromosomes and sample GC and the models is given in Supplementary files 6.

As in most available NIPT pipelines, the main qualifier for calling a trisomy in NIPTmer is the z-score of observed coverage of the chromosome of interest^5–8^. Thus, the quality of discrimination depends on the variance of respective coverages in control dataset. Although the linear model lowers the variance, the result remains stochastic with certain amount of uncertainty. The lower is the sequencing coverage, the higher is stochastic variability and the less powerful is the method. More detailed analysis of the variance at different coverage intervals is given in Supplementary files 7. The relative coverage (or z-score) distribution among euploid and aneuploid cases probably depends on both technological (cfDNA extraction method, sequencing library preparation, NGS technology, and equipment used) and biological (gestational age, overall health of mothers, population) factors. Thus, the actual cut-offs for calling the elevated risk of common autosomal trisomies should be defined in every NIPT laboratory separately. These cut-offs determine the predictive power and the proportions of false-negative and false-positive NIPT results. Setting cut-off value too stringently would minimize the number of false-positive results and eventually enables to reduce significantly the number of invasive prenatal tests, which is one of the issues addressed by NIPT. However, it should be kept in mind that this will introduce a number of false-negatives that must be avoided. Therefore, defining cut-off values for calling the elevated risk of fetal aneuploidy is critical. The importance of choosing suitable cut-offs in every separate NIPT laboratory can be illustrated by our study, where different cut-off values should be applied for Estonian and Belgian cohorts. Overall, in our case, the relatively low discriminating power in Belgian cohort associated with lower z-scores of trisomy samples when compared to Estonian trisomy samples and thus the need to adjust cut-off values, could be explained by several factors, including approximately two-fold lower coverage (0.32 vs 0.13) and shorter reads length (85 bp vs 50 bp) compared to Estonian cohort. In addition, it cannot be excluded that the reference group of Estonian origin used for analysis of both, Estonian and Belgian, cohorts, did not suit the best in the latter case and could influence the results obtained for the Belgian cohort.

NIPTmer also gives the estimation of sequencing uniformity in the form of Mahalanobis distance of a given sample. High values of this parameter indicate that sample is not typical, either because it has chromosomal aneuploidy or there was some other cause of variance between the relative sequencing coverage of different chromosomes. We did not use this value in our validation due to too small sample sizes, but in the future, it can be used to indicate the need of re-sequencing or further study.

Another thing that was not considered in the current study is fetal fraction (FF) or the proportion of fetal cfDNA to the total cfDNA of a sample. Because cfDNA is a mixture of both maternal and fetal cfDNA, the ability to detect fetal chromosomal aneuploidies is directly related to FF. Too low FF (<4%, a common minimum threshold) may give rise to a false negative result, as the difference in the ratio of an aberrant chromosome would be insufficient to distinguish^10^. Therefore, it is important to estimate FF accurately, although currently there is a lack of standardisation in this field^11^. Several approaches for FF calculation have been proposed that differ by the target domain of the fetal-derived sequence being measured by the assay, specificity, reproducibility and the cost^11,12^. However, no large-scale comparison between different measurement methods has been done, therefore none of them can be considered a “standard” FF measurement^11^. Current NIPTmer version does not calculate FF, although this step should be incorporated into the analysis pipeline in the future. One possibility is to use SeqFF approach that is based on the principle of size fractionation and does not require any additional experiments while calculating FF from shallow-depth single-end sequencing data^13^.

Although the discriminative power of NIPT can probably be further increased, it cannot completely replace ultrasound screening and cytogenetic analyses in the near future. The known weak spot of NIPT is associated with possible fetal mosaicism. As the fetal cfDNA, in fact, originates from the placenta, NIPT will miss the cases where placenta is euploid while fetus is fully or partially aneuploid, resulting in false negatives. For example, a mosaic T13 sample from the validation dataset was not detected with our pipeline. Also, the mosaic T18 in our test data had significantly lower z-score compared to full trisomy cases. Although setting the cut-off at 3.5 SD would enable to discriminate this specific mosaic T18 case in our dataset, we expect that this would also increase a number of false positive results in future tests. After all, NIPT is a method for evaluating the genotype, but the ultimate goal of prenatal testing is to determine the phenotype of fetus.

In summary, NIPTmer is an innovative tool for the ultra-rapid analysis of NIPT data. NIPTmer replaces time-consuming sequencing read mapping with a resource-saving k-mer counting strategy. In addition to its high performance, NIPTmer provides accurate and robust detection of common autosomal fetal trisomies, as was demonstrated in two independent cohorts.

## Methods

### Samples

NIPTmer was tested using two sets of cfDNA samples of pregnant women, one from Estonia and another from Belgium. The Estonian sample set consisted of 294 samples and was used to train NIPTmer algorithm. Sequencing libraries were generated using Illumina TruSeq ChIP Sample Preparation kit (Illumina Inc., San Diego, CA, USA) and sequenced on Illumina NextSeq 500 instrument with an average coverage of 0.32 × (minimum 0.08, maximum 0.42) producing 85 bp single-end reads. The Belgian dataset, published previously^3^, consisted of 289 samples and was used in a blind test to validate the algorithm by comparing the results of the two pipelines. Libraries were prepared using TruSeq ChIP Sample Preparation kit (Illumina) and sequenced on Illumina HiSeq 2500 producing 50 bp single-end reads with an average coverage of 0.13x^3^.

The study was approved by Research Ethics Committee of the University of Tartu (#246/T-21) and written informed consent was obtained from all participants. All experiments were performed in accordance with relevant European guidelines and regulations.

### NIPTmer pipeline

NIPTmer pipeline consists of three main steps: (1) creating k-mer lists, (2) counting the k-mers from the samples, and (3) calling the aneuploidy based on the counts.

### Step 1: Creation of k-mer lists

**Figure 4:**
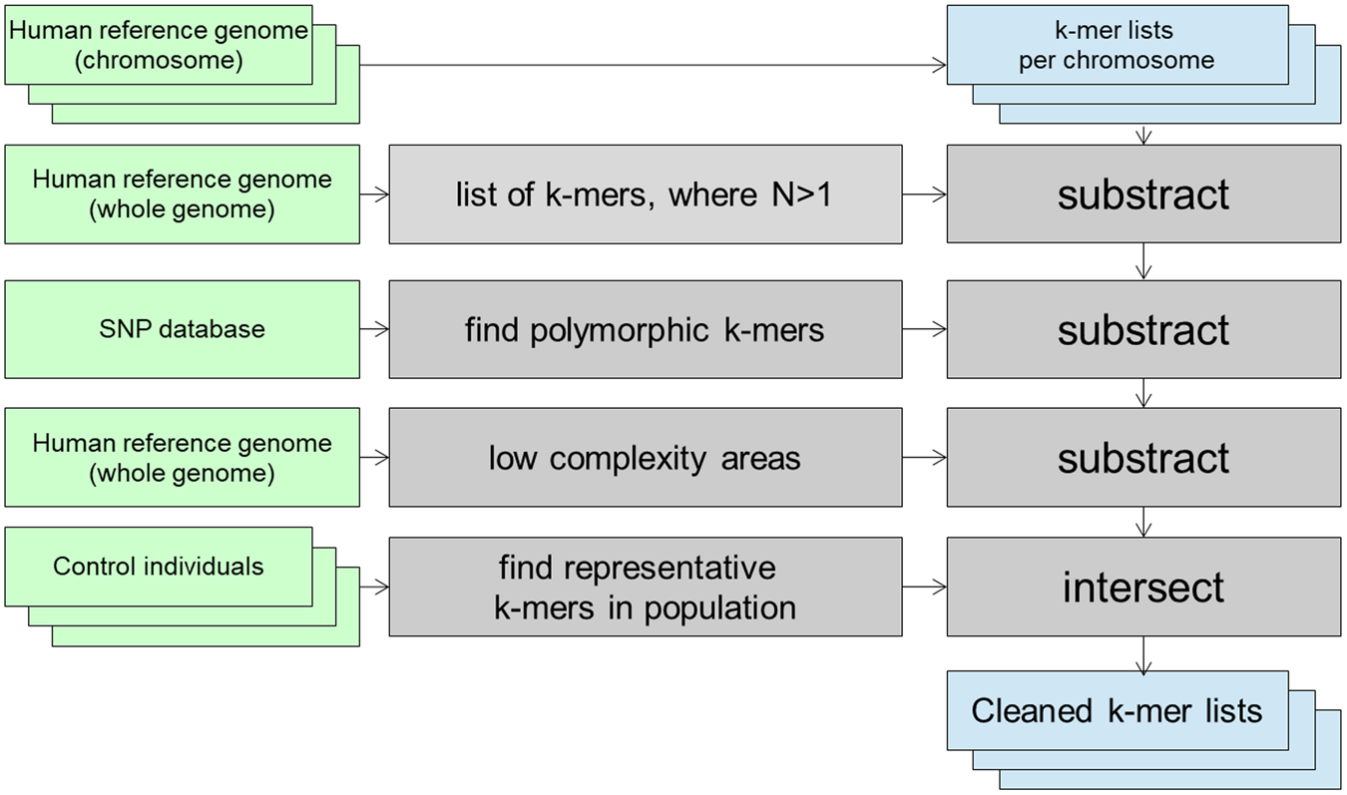
Creation of per-chromosome k-mer lists is composed of the following steps: creating lists of per-chromosome k-mers from reference genome; removing non-unique k-mers; removing potentially polymorphic k-mers; removing k-mers from problematic regions of genome like centromeres, telomeres, pseudoautosomal regions etc; and intersecting with list of k-mers that have normal copy number in population.

Creating k-mer lists from FASTQ files and conducting intersection and subtraction operations with the k-mer lists was done using GenomeTester4 toolkit^14^. Figure 4 gives the overview of the creation of k-mer lists.

The first step is a multistage process in which a list of unique reference k-mers is generated for each chromosome. The initial list containing all possible k-mers in a given chromosome was refined by excluding (subtracting) certain groups of k-mers and intersecting it with a list of stable k-mers. In the first stage, the list of non-unique k-mers was subtracted from the initial chromosome-specific k-mer lists to ensure that only unique k-mers are included in the final step. In the second stage, k-mers that overlap with any allele of known polymorphisms were removed to ensure that the list is universal for all individuals and ethnic groups. Specifically, a FASTA file was generated containing regions with possible allele combinations of known polymorphisms in dbSNP with MAF above 1%. All k-mers in obtained file were removed from chromosome-specific k-mer lists. In our analysis, the human reference genome version GRCh37 and dbSNP polymorphism database version 147 was used.

Low-complexity repeats are known to cause variability in sequencing data and, therefore, should be excluded from the final k-mer lists. In the third stage, known low-complexity repeats, including telomeric and centromeric regions, and pseudoautosomal regions were removed by constructing FASTA files containing known problematic regions and subtracting all k-mers of obtained file from chromosome-specific k-mer lists. The low complexity regions were downloaded from 1000 Genomes project.

Finally, full-genome sequencing data of euploid control individuals were used to obtain a list of k-mers that have the expected frequency distribution in the control population. This list enables to account for sequencing bias, contamination, copy number variations, and other unaccounted sources of k-mer variation. Sequencing of control and NIPT samples should be performed on the same or similar platforms, but the coverage for control samples must be significantly higher.

The numbers of reads from each genomic location in control samples, and thus the observed number of copies of each unique k-mer, were approximated with a Poisson distribution with the mean being the average sequencing depth. First, a raw k-mer list was constructed from the sequencing reads of each control individual. High and low cut-off values of k-mer counts were calculated based on the cumulative Poisson distribution, with the actual sequencing coverage as a mean. The probability of the observed k-mer count may be too low, and the k-mer unsuitable for further workflow, if (i) the given k-mer is not present in exactly two copies in a given genome or (ii) the given part of genome has strong sequencing bias. For each control individual, a list of k-mers was constructed with a count between the calculated cut-off values. In the current study, 50 control samples from EGCUT^15^ were used with the probability cut-offs 0.01 and 0.99. Sequencing of control individuals was performed on Illumina HiSeq 2500 system with coverage of 20–30x. The intersection of all per-individual lists gave a per-population list of stable k-mers. By intersecting this list with per-chromosome k-mer lists, final filtered per-chromosome lists were obtained.

Detailed description of each step can be found in supplementary materials (Supplementary files 5, section A).

### Step 2: Counting sample-specific k-mers

First, the sequencing reads in raw FASTQ files of a sample were converted to 25-mer list. The obtained k-mer list was intersected one-by-one with all chromosome-specific filtered k-mer lists and thus, the number of k-mers specific to each chromosome in a particular sample (*K*_*sc*_) was found. In addition, the average GC content of sequenced reads (*GC*_*S*_) was calculated. All operations in this step were performed using GenomeTester4 toolkit^14^. The details are available in the supplementary materials (Supplementary files 5, section B).

### Step 3: Coverage model for aneuploidy calling

The input for risk score calculation section was a data-matrix containing raw count of unique k-mers for each sample and each chromosome, as well as the number on k-mers in the reference k-mer lists and GC content of the sequences.

Aneuploidy of a certain chromosome was called based on the increase or decrease of its coverage compared to expected value for this sample. These changes are usually small, because the major part of cfDNA originates from mother’s euploid cells. If n% of the total cfDNA is of fetal origin, then, in case of full non-mosaic trisomy, the relative change of coverage of one chromosome is (Equation 1):

1 $$(100+\frac{3}{2}{\rm{n}})\div(100+{\rm{n}})-1$$

Thus, a good prediction of expected coverage is crucial in order to detect minor changes in coverage.

Per-sample per-chromosome coverage (*C*_*sc*_) was calculated by dividing the k-mer count by the k-mer list length (*L*_*c*_) of a given chromosome (Equation 2):

2 $${{\rm{C}}}_{{\rm{SC}}}=\frac{{{\rm{K}}}_{{\rm{SC}}}}{{{\rm{L}}}_{{\rm{C}}}}$$

*C*_*sc*_ – per-sample per-chromosome coverage

*K*_*sc*_ – number of chromosome-specific k-mers in a sample

*L*_*C*_ – total number of chromosome-specific k-mers in k-mer list

According to our analysis, the relative coverages *C*_*sc*_ of different chromosomes varied even in euploid samples. The effect, which is mostly consistent between samples, depended on the GC content of a chromosome, GC content of a sample and possibly on other factors as well. Because of this variance between the samples, the average sequencing coverage could not be reliably used and more precise linear model was constructed^16^ (Equation 3):

3 $$\hat{\beta }={({{\rm{X}}}^{{\rm{T}}}{\rm{X}})}^{-1}{{\rm{X}}}^{{\rm{T}}}{\rm{y}}$$

*β* – regression coefficient vector

*X* – coverage matrix *C*_*sc*_ excluding the parameter at hand (chromosome or GC)

*y* – vector of values of given parameter

The regression coefficients calculated in the previous step were used to predict expected per-chromosome coverages of a new sample (Equation 4):

4 $${\rm{C}}{^{\prime} }_{{\rm{SC}}}{={\rm{\beta }}}_{1}{{\rm{C}}}_{1}{+{\rm{\beta }}}_{2}{{\rm{C}}}_{2}+\mathrm{..}.{+{\rm{\beta }}}_{22}{{\rm{C}}}_{22}{+{\rm{\beta }}}_{{\rm{GC}}}{\rm{GC}}$$

*C*′_*SC*_ – predicted per-sample per-chromosome coverage

*β*_1_*… β*_22_, *β*_*GC*_ – model coefficients

*GC* – the average GC content of a sample

Separate linear regression models were built for each chromosome so that *C*_*sc*_ of a given chromosome was the dependent variable and all other chromosomal coverages and the GC content of the sample were independent variables. Expected coverages (*C*′_*sc*_) of chromosomes were calculated based on those models for each sample in analysis.

To determine the sex of a fetus, four sex chromosome models (X female, X male, Y female and Y male) were created to predict the expected coverages of sex chromosomes based on autosome coverage. Model parameters were estimated using a reference dataset of 150 euploid pregnancies. The model for autosomes was calculated using all reference samples. For sex chromosome models, only samples from pregnancies with fetus of the corresponding sex were used.

By applying these models on an unknown sample, the expected coverages of all chromosomes were obtained, assuming that the sample was similar to the reference (i.e. euploid). For sex chromosomes, four expectation values were obtained – two based on the assumption that the fetus was a male and two on the assumption that it was a female.

The difference between predicted and actual per-chromosome coverage was expected to have normal distribution, given uniform sequencing depth. To make the algorithm less dependent on sequencing depth, a difference can be normalized by dividing the actual difference with the observed per-chromosome coverage (Equation 5):

5 $${{\rm{D}}}_{{\rm{SC}}}=\frac{{{\rm{C}}}_{{\rm{SC}}}-{\rm{C}}{^{\prime} }_{{\rm{SC}}}}{{\rm{C}}{^{\prime} }_{{\rm{SC}}}}$$

*D*_*sc*_ – normalized difference between observation and prediction

*C*_*sc*_ – per-sample per-chromosome coverage

*C*′_*SC*_ – predicted per-sample per-chromosome coverage

Samples were analysed by standardizing (z-scoring) the difference of the per-chromosome coverage and the predicted coverage (*D*_*sc*_) for the same chromosome. For standardization, the data of reference group was used to find the mean difference and the standard deviation (Equation 6):

6 $${{\rm{Z}}}_{{\rm{SC}}}=\frac{{{\rm{D}}}_{{\rm{SC}}}-{\rm{average}}({{\rm{D}}}_{\text{SC},\text{ref}})}{{\rm{SD}}({{\rm{D}}}_{\text{SC},\text{ref}})}$$

*Z*_*SC*_ – Z-score of model prediction for specific sample and chromosome

*D*_*sc*_ – difference between observation and prediction

*D*_*sc*,*ref*_ – difference between observation and prediction of reference population

The z-score was used as the indicator of aneuploidy risk for a certain chromosome. The practical z-score cut-off values, indicating the elevated risk for fetal aneuploidies, depend on the reference and test populations, sequencing technology, and coverage used. Therefore, these values must be determined experimentally.

The Mahalanobis distance of a given sample from the reference set can be used to estimate DNA preparation and sequencing quality (Equation 7):

7 $${D}_{M}({\vec{C}}_{S})=\sqrt{{({\vec{C}}_{S}-\vec{C})}^{T}{S}^{-1}{({\vec{C}}_{S}-\vec{C})}^{T}}$$

*D*_*M*_ – Mahalanobis distance

${\vec{C}}_{S}$– vector of all normalized per-chromosome coverages plus GC of analyzed sample

$\vec{C}$– vector of averages of per-chromosome coverages plus GC in control dataset

*S*^−1^ – covariance matrix of *C*_*SC*_ plus GC in control dataset

**Figure 5:**
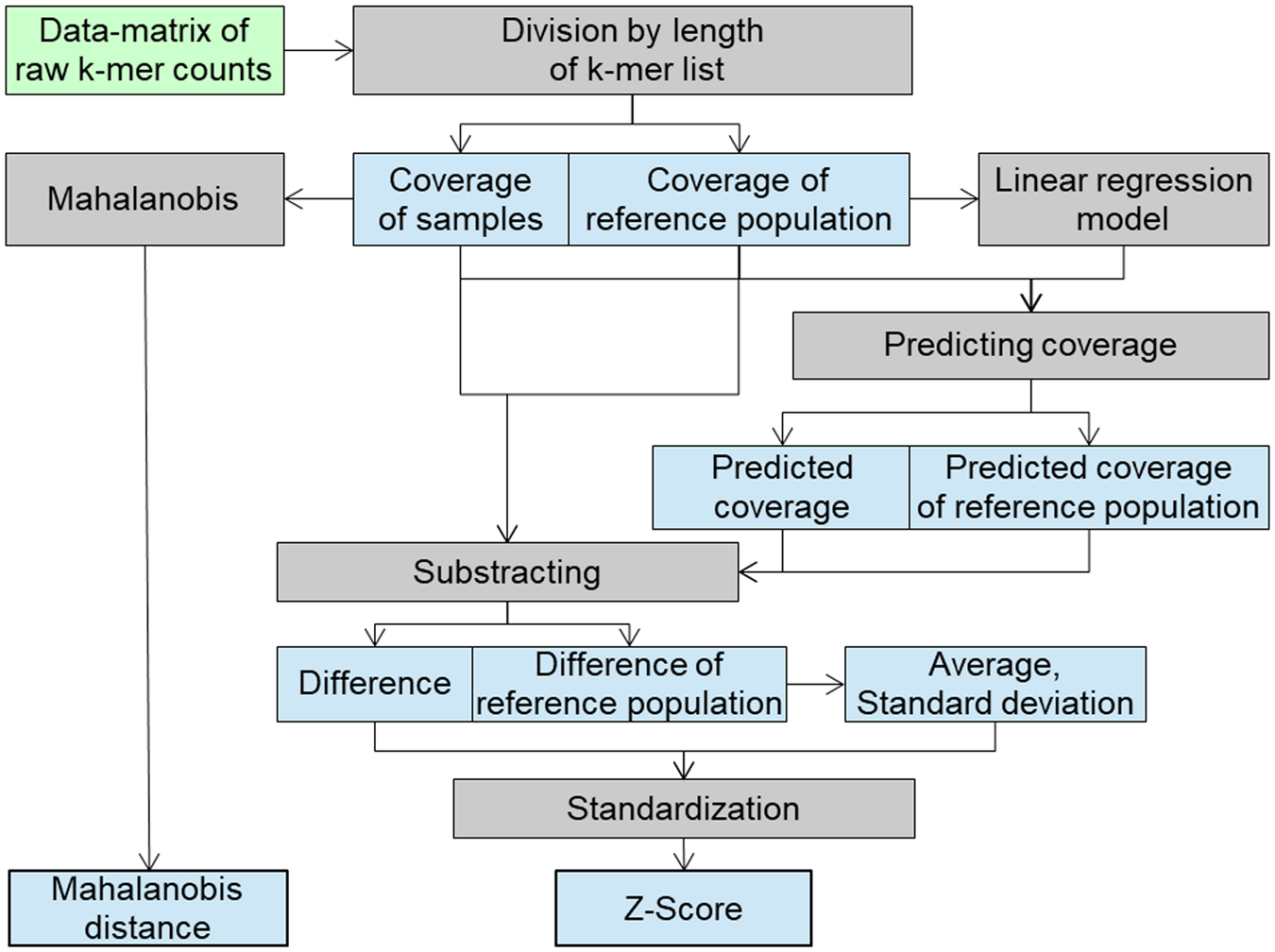
Data flow during aneuploidy calling step. Raw counts of each chromosome are divided by the length of k-mer list of respective chromosome to obtain the coverage. The coverage is separated between sample group and reference group. Only reference group is used to generate the linear regression model and later to calculate the average and standard deviation between the observed and predicted coverages. Z-score is used to call the aneuploidy. Cut-off is customizable depending on the type 1 and type 2 error tolerated in the study. Mahalanobis distance is used as a quality control parameter.

The workflow for aneuploidy calling step is summarized in Fig. 5. The details are available in the supplementary materials (Supplementary files 5, section C).

## Electronic supplementary material

Supplementary file 1

Supplementary file 2

Supplementary file 3

Supplementary file 4

Supplementary files 5, 6, 7
