# Supplementary material for: NIPTmer: rapid k-mer-based software package for detection of fetal aneuploidies
Source: Sci Rep. 2018 Apr 4;8:5616. doi: 10.1038/s41598-018-23589-8 (PMC5884839; doi:10.1038/s41598-018-23589-8)
Supplement: Supplementary file 5 — Supplementary files 5, 6, 7 [file 41598_2018_23589_MOESM5_ESM.pdf]

NIPTmer: rapid k-mer-based software package for detection of fetal aneuploidies

Martin Sauk[1], Olga Žilina[1], Ants Kurg[1], Eva-Liina Ustav[2,3], Maire Peters[2,4], Priit Paluoja[4], Anne Mari Roost[4], Hindrek Teder[4,5], Priit Palta[6], Nathalie Brison[7], Joris R. Vermeesch[7], Kaarel Krjutškov[4,8,9], Andres Salumets[2,4,5,10,+,*], Lauris Kaplinski[1,+,*]

[1] Institute of Molecular and Cell Biology, University of Tartu, Tartu, Estonia

[2] Department of Obstetrics and Gynaecology, Institute of Clinical Medicine, University of Tartu, Tartu, Estonia

[3] Women's Clinic, Tartu University Hospital, Tartu, Estonia

[4] Competence Centre on Health Technologies, Tartu, Estonia

[5] Department of Biomedicine, Institute of Bio- and Translational Medicine, University of Tartu, Tartu, Estonia

[6] Estonian Genome Center, University of Tartu, Tartu, Estonia

[7] Center for Human Genetics, KU Leuven, Leuven, Belgium

[8] Department of Biosciences and Nutrition, Karolinska Institutet, Huddinge, Sweden

[9] Molecular Neurology Research Program, University of Helsinki and Folkhälsan Institute of Genetics, Helsinki, Finland

[10] Department of Obstetrics and Gynecology, University of Helsinki and Helsinki University Hospital, Helsinki, Finland

+ Last co-authorship

* To whom correspondence should be addressed: andres.salumets@ccht.ee and lauris.kaplinski@ut.ee

Supplementary file 5

NIPTmer - a kmer based NIPT analysis pipeline

The pipeline has three basic steps:
A. Create reference kmer lists (to be run once before analysis).
B. Create control dataset.
C. Analyze samples.

NIPTmer uses GenomeTester4 programs to generate and modify k-mer lists.[1] It has to be downloaded and compiled before executing pipeline commands. By default all pipeline scripts expect to find the GenomeTester4 binaries (glistmaker, glistcompare and glistquery) in the current directory, but the location can be edited in script headers.

A. Creating reference kmer lists

A.1. Creating chromosome (or sub-chromosomal region) specific lists

Usage: `generate_reference_lists.pl WORDLENGTH FASTA_FILES...`

Creates a set of chromosome 'whitelists', i.e. GenomeTester4 lists of k-mers of specified length that occur once and only once in any given FASTA file. Usually the FASTA files are reference chromosomes.

Internally it executes the following steps:
1.1. Creates composite list of all k-mers that occur more than once in the set of all FASTA files, using glistmaker.
Then, for each FASTA file:
1.2. Creates list of all kmers in given file with glistmaker.
1.3. Subtracts the list created in 1.1. from chromosome-specific list file, using glistcompare diff function. Thus a list of unique kmers of given FASTA sequence is obtained.

The resulting lists are named
`BASENAME_WORDLENGHT.list`
Where BASENAME is the name of FASTA file, excluding directory path and extension (everything after '.').

A.2. Creating blacklist of known polymorphisms

Usage: `generate_polymorphism_lists.pl WORDLENGTH MIN_MAF PATH_TO_CHROMOSOME_FILE VCF_FILES...`

Creates list named plymorphic_blacklist.list that contains allele-specific k-mers that may potentially occur at the polymorphic sites with MAF >= cutoff.

Internally it executes the following steps:

1 Kaplinski L, Lepamets M, Remm M. (2015). GenomeTester4: a toolkit for performing basic set operations – union, intersection and complement on k-mer lists. GigaScience; 4:58

For all VCF files:

2.1. Creates FASTA file of pseudo-haplotypes (i.e. all potential haplotypes ignoring linkage) that overlap with known polymorphisms. To conserve space, SNPs are marked with UIPAC codes.
Usage: `hapl_generator.pl WORDLENGTH MIN_MAF VCF_FILE CHROMOSOME_FILE`

2.2. Generates FASTA file of all kmers occurting in given pseudo-haplotype file. For each IUPAC code all potential permutations are created
`kmer_creator.pl WORDLENGTH HAPLOTYPE_FILE`

Then:

2.3. Create list of all k-mers in all k-mer FASTA files using glistmaker

2.4. Cut all k-mer frequencies to 1 using glistcompare

It uses two sub-scripts: `hapl_generator.pl` and `kmer_creator.pl`

The resulting list is named:
`polymorphic_blacklist_WORDLENGTH.list`

A.3. Creating backlist from BED files

Known problematic regions of genome, such as low-complexity repeats and pseudoautosomal parts of sex chromosomes can be compiled into separate ‘blacklist’ from corresponding BED files.

3.1. Create FASTA file of problematic sequences.

Usage: `bed-2-fasta.pl GENOME BED > FASTA`

`GENOME` is FASTA file of the whole genome, BED lists regions to be included in blacklist FASTA file (excluded from k-mer sets).

3.2. Create blacklist

Blacklist is compiled with glistmaker

Usage: `glistmaker FASTA -w WORDLENGTH -o lc_blacklist`

The resulting blacklist is named `lc_blacklist_WORDLENGTH.list`

A.4. Creating ‘whitelists’ using sequencing data

Individual BAM files or GenomeTester4 lists of full-genome sequencing data can be compiled into individual ‘whitelists’ with script:

Usage: `poisson_cutoff.pl LIST|BAM COVERAGE CUTOFF [haploid]`

Creates a list of all k-mers in source list/bam file that have the probability greater or equal to cutoff value of coming from Poisson distribution with mean coverage. If 'median' is used in place of coverage, the coverage is automatically calculated. If the argument "haploid" is present, the haploid coverage value is used to calculate cumulative Poisson probabilities of each k-mer count. This should be used when creating ‘whitelist’ for sex chromosomes from male samples.

Internally it executes the following steps:

4.1. If the file extension is .bam, a kmer list is generated from BAM file using script

bam2list.pl.

4.2. If coverage value is 'median', the coverage is estimated by finding the first peak in k-mer frequencies above 1.

4.3. Cumulative Poisson cutoff values MIN and MAX are calculated at `CUTOFF` and `1-CUTOFF.`

4.4. Temporary list A of k-mers with frequency less than MIN is created from full list with glistcompare intersection function.

4.5. Temporary list B with k-mer frequencies above MAX is created from list A with glistcompare intersection function.

4.6. List B is subtracted from list A with glistcompare diff function.

The resulting list is named
`BASENAME_whitelist_WORDLENGTH.list`
Where BASENAME is the name of list/BAM file, excluding directory path and extension (everything after '.').

4.7. Multiple ‘whitelists’ can be combined into single whitelist with glistcompare intersection method:

Usage: `glistcompare LISTS... -i -o whitelist`

The resulting whitelist is named `whitelist_WORDLENGTH_intrsec.list`

NB! As the autosomes and sex chromosomes have different copy numbers, whitelists should be compiled differently for autosomes and sex chromosomes. From female samples single autosome and X ‘whitelists’ can be prepared. From male samples autosome and sex chromosome ‘whitelists’ should be compiled separately.
NB! Male and female sex-chromosome ‘whitelists’ should never be intersected (as described in 4.7) because as the female samples do not contain Y chromosome, all Y-specific k-mers will be missing in the result.

A.5. Creating final k-mer lists

‘Blacklists’ should be subtracted and ‘whitelists’ intersected with chromosome-specific k-mer lists created in step 1. This can be done with GenomeTester4 program glistcompare:

5.1. Subtracting blacklist:

Usage: `glistcompare LIST BLACKLIST -d -o NAME`

The resulting list is named `NAME_WORDLENGTK_0_diff1.list`

5.2. Intersecting with whitelist:

Usage: `glistcompare LIST WHITELIST -i -o NAME`

The resulting list is named `NAME_WORDLENGTH_intrsec.list`

B. Creating control dataset

B.1. Creating kmer lists from sequenced control samples

From each sequenced sample (fastq files) a GenomeTester4 k-mer list can be created with command:

Usage: `glistmaker FASTQ_FILES... -w WORDLENGTH -o NAME`

The resulting list in named `NAME_WORDLENGTH.list`

B.2. Calculate k-mer counts for each sequenced sample

Usage: `make_table.pl CHR_SUBDIR LISTS...`

`CHR_SUBDIR` is directory, where chromosome-specific k-mer lists are stored. By default it looks it for all files named `CHRID_cleaned_25.list` (`CHRID` is one of 1, 2, 3...23, X, Y). Both the chromosome names and list suffix can be changed in script header.

This script a table with chromosomes as columns and samples as rows showing how many k-mers from each chromosome-specific list was present in sequenced data. The first row after header is the total k-mer counts in chromosome-specific lists.

Internally the script uses glistcompare to find the intersection size of two lists - sample list and chromosome list.

The table has the following rows:
CHR - chromosome names;
TOTAL - total number of k-mers in chromosome-specific list;
GC - average GC content of all chromosome-specific k-mers;
Then one row for each sequenced sample.

The table has following columns:
CHR (first column) - sample name;
TOTAL - total number of k-mers in sample;
UNIQUE - number of unique k-mers in sample;
GC - average GC content of sample;
Then one column for each chromosome.

Each entry of table (aside headers, GC content etc.) is the number of k-mers specific to certain chromosome (column) in certain sample (row). If only some samples are to be added to already existing dataset, the script add_rows.pl can be used:

Usage: `add_rows.pl CHR_SUBDIR LISTS...`

Its parameters and output are identical to make_table.pl, but it does not print out header row. Thus it can be used to append rows to already existing table.

C. Analyzing samples

Both control and patient k-mer counts should be listed in single table (prepared in step B).
Then the program ZandMah.py is used to calculate z-scores:

Usage: `ZandMah.py -i KMER_TABLE -r CONTROLS -n -o OUTPUTFILE`

`KMER_TABLE` is the full table of sample k-mer counts prepared in step B.

`CONTROLS` is text file listing all control samples (i.e. euploid samples) for model calculation. Each sample-name on a separate row. Option to add known genders "male" or "female", separated from sample-name with a tab.

`-n` is a flag that allows to swich normalization on or off. Adding this flag to the command line swiches normalization on.

The output is table, where rows are samples and columns chromosomes. Each entry corresponds to z-score of given chromosome k-mer count, i.e. values above certain threshold mark possible aneuploidy. In addition the Mahalanobis distance of given sample and predicted sex is printed as last columns.

Supplementary file 6

Relations between chromosomes

As one can expect, the relative (normalized by total coverage) k-mer counts of different chromosomes vary between samples. Although certain part of this variance is certainly stochastic and caused by the stochastic nature of DNA fragmentation and sequencing, there are certain patterns – i.e. the relative counts of different chromosomes are correlated (Table S6.1).

Table S6.1
Pearson correlation coefficients between the normalized coverages of different chromosomes. Chromosomes are ordered by the average GC content of respective k-mer lists (values in second row and second column). GC_corr is the correlation between normalized coverage of given chromosome and sample GC content.

CHR	GC	GC corr	4	13	6	3	5	18	8	2	12	7	14	10	21	9	1	15	11	20	16	17	22	19
			0,400	0,404	0,415	0,416	0,416	0,418	0,422	0,423	0,426	0,427	0,430	0,437	0,437	0,438	0,439	0,440	0,442	0,462	0,467	0,476	0,502	0,505
4	0,400	-0,98	1	1,00	0,98	0,98	0,99	0,96	0,97	0,97	0,94	0,87	0,94	-0,15	0,23	0,14	-0,66	-0,66	-0,79	-0,99	-0,98	-0,99	-0,99	-0,95
13	0,404	-0,98	1,00	1	0,98	0,97	0,98	0,96	0,97	0,97	0,94	0,86	0,93	-0,16	0,24	0,13	-0,66	-0,66	-0,79	-0,98	-0,98	-0,99	-0,98	-0,95
6	0,415	-0,97	0,98	0,98	1	0,99	0,99	0,96	0,97	0,99	0,97	0,84	0,94	-0,04	0,21	0,18	-0,54	-0,55	-0,76	-0,97	-0,99	-0,98	-0,99	-0,98
3	0,416	-0,96	0,98	0,97	0,99	1	0,99	0,96	0,97	0,98	0,97	0,84	0,94	-0,05	0,20	0,18	-0,54	-0,55	-0,76	-0,96	-0,98	-0,98	-0,99	-0,98
5	0,416	-0,96	0,99	0,98	0,99	0,99	1	0,97	0,98	0,98	0,96	0,86	0,94	-0,09	0,20	0,15	-0,62	-0,61	-0,76	-0,97	-0,97	-0,99	-0,99	-0,97
18	0,418	-0,94	0,96	0,96	0,96	0,96	0,97	1	0,97	0,95	0,94	0,83	0,92	-0,06	0,18	0,17	-0,60	-0,56	-0,74	-0,94	-0,95	-0,98	-0,97	-0,96
8	0,422	-0,94	0,97	0,97	0,97	0,97	0,98	0,97	1	0,97	0,95	0,86	0,92	-0,06	0,18	0,15	-0,61	-0,59	-0,72	-0,95	-0,96	-0,99	-0,98	-0,97
2	0,423	-0,96	0,97	0,97	0,99	0,98	0,98	0,95	0,97	1	0,95	0,84	0,93	0,03	0,20	0,19	-0,51	-0,54	-0,72	-0,95	-0,98	-0,98	-0,98	-0,98
12	0,426	-0,92	0,94	0,94	0,97	0,97	0,96	0,94	0,95	0,95	1	0,80	0,92	-0,04	0,14	0,18	-0,50	-0,49	-0,71	-0,93	-0,95	-0,96	-0,97	-0,95
7	0,427	-0,84	0,87	0,86	0,84	0,84	0,86	0,83	0,86	0,84	0,80	1	0,79	-0,14	0,19	0,08	-0,61	-0,63	-0,68	-0,85	-0,86	-0,88	-0,86	-0,84
14	0,430	-0,93	0,94	0,93	0,94	0,94	0,94	0,92	0,92	0,93	0,92	0,79	1	-0,10	0,15	0,17	-0,55	-0,53	-0,76	-0,92	-0,93	-0,94	-0,94	-0,92
10	0,437	0,12	-0,15	-0,16	-0,04	-0,05	-0,09	-0,06	-0,06	0,03	-0,04	-0,14	-0,10	1	-0,14	0,20	0,52	0,51	0,22	0,17	0,06	0,08	0,08	-0,08
21	0,437	-0,28	0,23	0,24	0,21	0,20	0,20	0,18	0,18	0,20	0,14	0,19	0,15	-0,14	1	-0,05	-0,28	-0,27	-0,36	-0,28	-0,26	-0,23	-0,22	-0,22
9	0,438	-0,18	0,14	0,13	0,18	0,18	0,15	0,17	0,15	0,19	0,18	0,08	0,17	0,20	-0,05	1	0,13	0,10	-0,12	-0,15	-0,18	-0,15	-0,16	-0,24
1	0,439	0,60	-0,66	-0,66	-0,54	-0,54	-0,62	-0,60	-0,61	-0,51	-0,50	-0,61	-0,55	0,52	-0,28	0,13	1	0,81	0,60	0,63	0,54	0,63	0,60	0,47
15	0,440	0,62	-0,66	-0,66	-0,55	-0,55	-0,61	-0,56	-0,59	-0,54	-0,49	-0,63	-0,53	0,51	-0,27	0,10	0,81	1	0,57	0,65	0,57	0,61	0,58	0,47
11	0,442	0,81	-0,79	-0,79	-0,76	-0,76	-0,76	-0,74	-0,72	-0,72	-0,71	-0,68	-0,76	0,22	-0,36	-0,12	0,60	0,57	1	0,80	0,76	0,76	0,76	0,72
20	0,462	0,98	-0,99	-0,98	-0,97	-0,96	-0,97	-0,94	-0,95	-0,95	-0,93	-0,85	-0,92	0,17	-0,28	-0,15	0,63	0,65	0,80	1	0,97	0,97	0,96	0,94
16	0,467	0,98	-0,98	-0,98	-0,99	-0,98	-0,97	-0,95	-0,96	-0,98	-0,95	-0,86	-0,93	0,06	-0,26	-0,18	0,54	0,57	0,76	0,97	1	0,97	0,97	0,97
17	0,476	0,96	-0,99	-0,99	-0,98	-0,98	-0,99	-0,98	-0,99	-0,98	-0,96	-0,88	-0,94	0,08	-0,23	-0,15	0,63	0,61	0,76	0,97	0,97	1	0,99	0,97
22	0,502	0,96	-0,99	-0,98	-0,99	-0,99	-0,99	-0,97	-0,98	-0,98	-0,97	-0,86	-0,94	0,08	-0,22	-0,16	0,60	0,58	0,76	0,96	0,97	0,99	1	0,97
19	0,505	0,95	-0,95	-0,95	-0,98	-0,98	-0,97	-0,96	-0,97	-0,98	-0,95	-0,84	-0,92	-0,08	-0,22	-0,24	0,47	0,47	0,72	0,94	0,97	0,97	0,97	1

One clear pattern one can immediately notice is that there is clear correlation between the sample GC content and the relative abundance of GC-rich chromosomes. As a consequence, the chromosomes with similar extreme GC content have positive correlation and chromosomes with different extreme GC content strong negative one. For example chromosomes 3, 4, 5 and 6 have low GC content and their coverages are strongly positively correlated. Chromosomes 16, 17, 19 and 20 have high GC content, they have strong positive correlation between themselves and strong negative one with 3, 4, 5 and 6.

GC content does not exhaust all the correlation between relative coverages. We calculated the expected coverages of different chromosomes in sample using the hypothesis that the relative probability of seeing certain DNA fragment is determined linearly by its GC content and the total GC content of a sample (linear GC model).

$$C_{chr}^{EXPECTED} = C_{sample}\left[GC_{sample}\frac{N_{chr}(GC_{chr})}{N_{genome}(GC_{genome})}(1-GC_{sample})\frac{N_{chr}(1-GC_{chr})}{N_{genome}(1-GC_{genome})}\right]$$

C_{sample} – total number of kmers in sample
$GCsample$ – sample GC content
N_{chr} – total number of chromosome specific k-mers
GC_{chr} – GC content of chromosome-specific k-mers
N_{genome} – total number of specific k-mers in genome

GC_{genome} – total GC content of all specific k-mers

The results can be seen in figure S6.1 and S6.2.

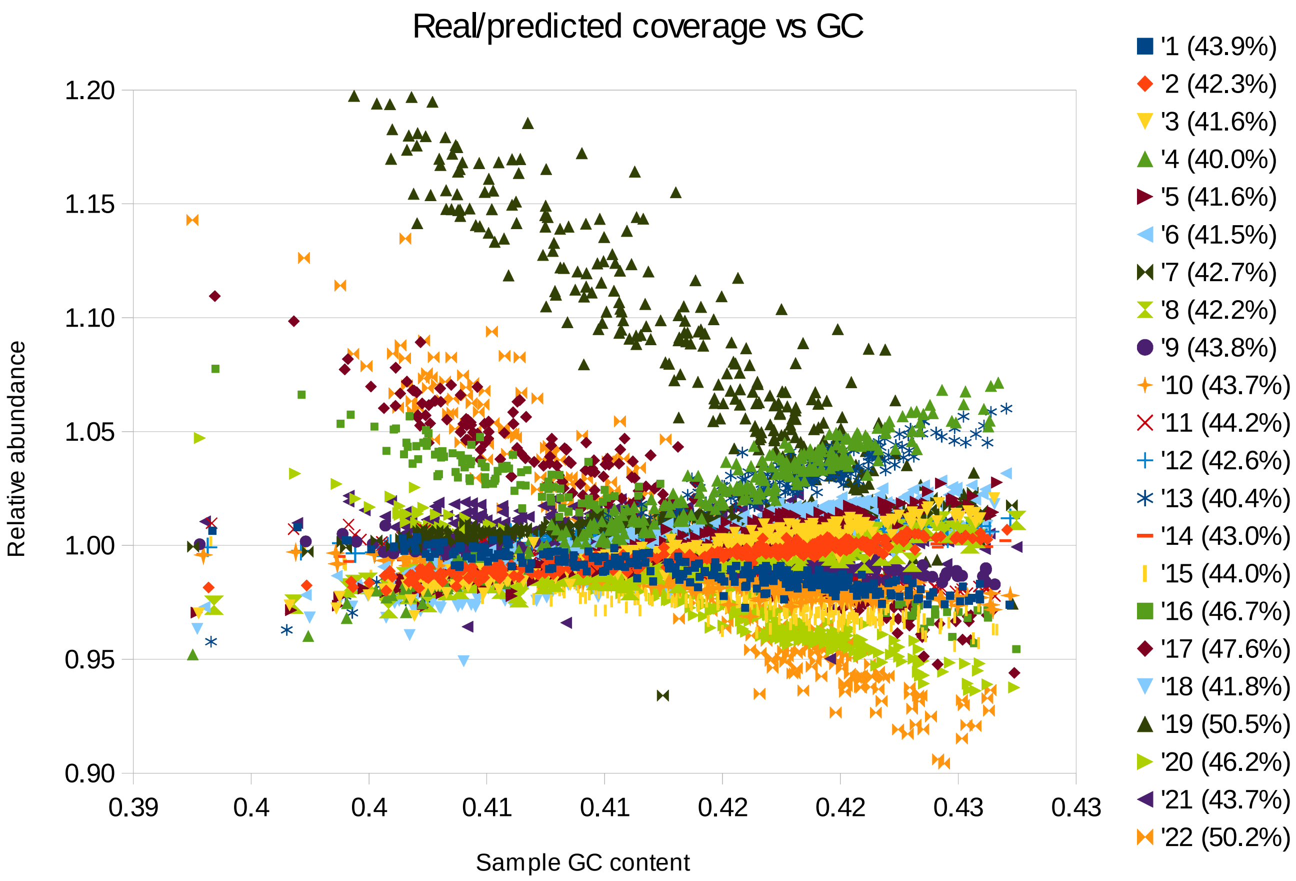

Figure S6.1. The relative difference (ratio) between predicted and actual coverage of different chromosomes depending on GC content of a sample.
X axis – the GC content of a sample. Y axis the relative difference (ratio) of actual and expected coverage of different chromosomes. GC content of per-chromosome k-mer list is listed next to chromosome name.

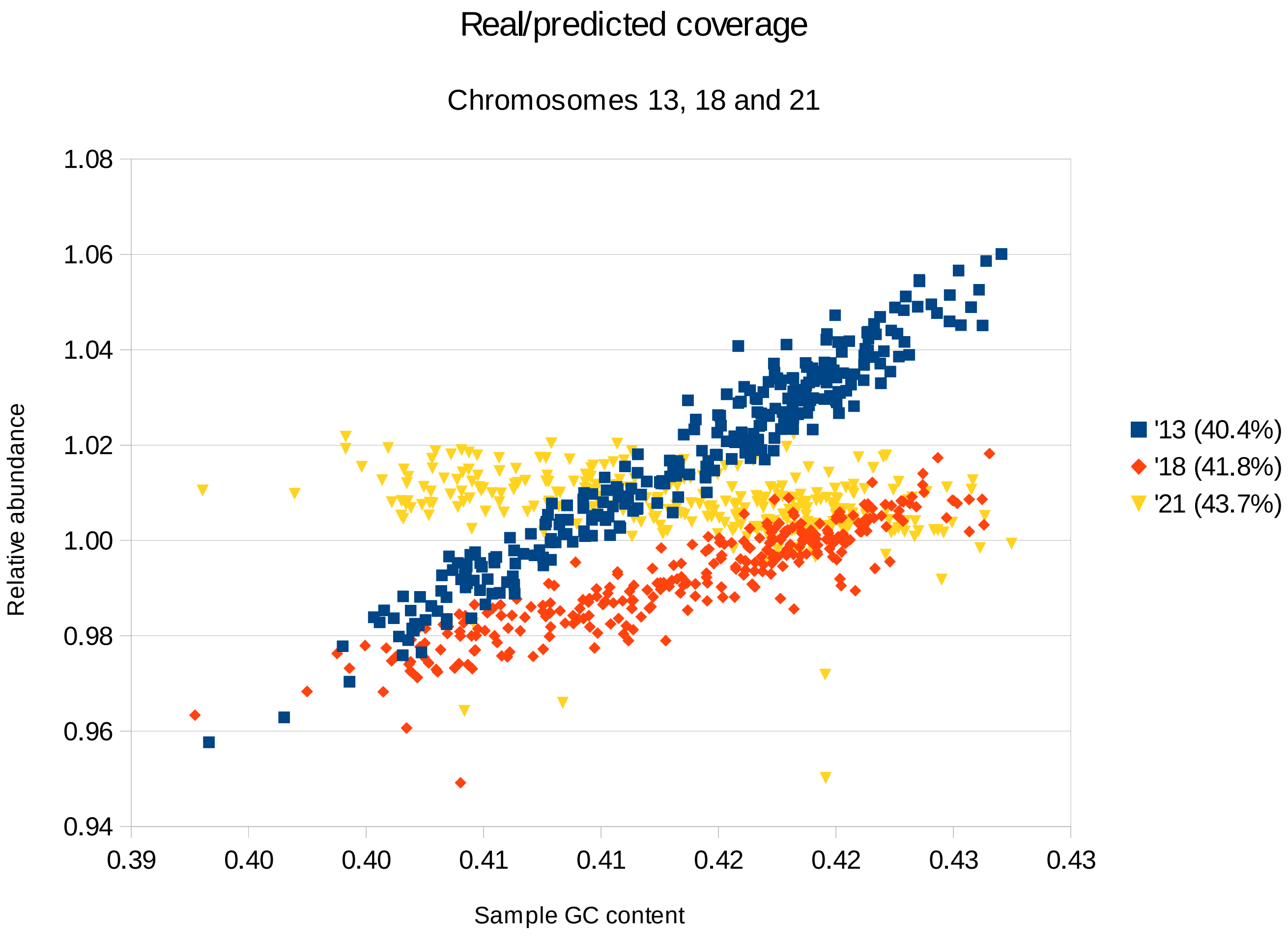

Figure S6.2. The relative difference between expected and actual coverage of chromosomes 13, 18 and 21 depending on GC content of a sample.
X axis – the GC content of a sample. Y axis the relative difference (ratio) of actual and expected coverage of different chromosomes. GC content of per-chromosome k-mer list is listed next to chromosome name.

As we can see, the linear GC model slightly overestimates the correlation of actual coverage and GC content. In high GC samples the chromosomes with low GC content are slightly overrepresented and the ones with high GC content slightly underrepresented. This implies, that the dependence of chromosome coverage and GC content is more complex.
More importantly, some chromosomes have additional effects, not described by model. For example, chromosome 16 and 20, although having similar GC content (and similar slope on figure) are separated by shift. The same for chromosomes 19 and 22 (Fig S 6.1). Although the slopes in general correlate with GC content, the absolute positions are different and do not intersect at single point.

To analyze the coverages more precisely we built linear models in R package to predict the relative coverage of different chromosomes, based on 4 sets of independent variables:
1. The average coverage of the sample
2. The average coverage of the sample + GC content of the sample
3. The relative coverages of all other autosomes
4. The relative coverages of all other autosomes + GC content of the sample

Models were built separately for chromosomes 13, 18 and 21 and evaluated by Akaike's An Information Criterion (AIC) and adjusted r^2 on the test dataset.

Chromosome 21

	df	AIC	Rsq adj
model_avg	3	-2000.169	0.9918
model_avg_gc	4	-2011.711	0.9922
model_all	23	-1975.757	0.9917
model_all_gc	24	-1974.420	0.9917

Chromosome 18

	df	AIC	Rsq adj
model_avg	3	-1695.069	0.9715
model_avg_gc	4	-2118.603	0.9932
model_all	23	-2376.193	0.9973
model_all_gc	24	-2374.723	0.9973

Chromosome 13

	df	AIC	Rsq adj
model_avg	3	-1417.854	0.9142
model_avg_gc	4	-2143.347	0.9927
model_all	23	-2622.080	0.9986
model_all_gc	24	-2629.192	0.9987

As one can see, the preferred model depends on chromosome. We decided to use the richest (the one with most parameters) model for the following reasons:
1. Richer models worked better for both chromosome 13 and 18.
2. Even if the models with fewer parameters work slightly better on test dataset for chromosome 21 we expect that if the dataset grows the difference either vanishes or reverses to the advantage of richer model. Using only the average coverage does not contain any additional information compared to the coverages of individual chromosomes and thus the AIC penalty of richer models is caused by larger number of parameters which penalizes small sample sets.
3. The difference between simple and rich models was much smaller for chromosome 21 than it was for other two chromosomes.

The richest model (GC and relative coverages of all other chromosomes) was implemented in final program.

Supplementary file 7

The effect of sequencing coverage

Due to the stochastic nature of sequencing we expect that the sequencing coverage influences the quality of NIPTmer predictions. At low coverage the stochastic variability of k-mer counts increases, rising the probabilities of both false negatives and positives.
The usable minimum sequencing coverage has to be determined by taking several variables into account:
1. The expected fetal fraction in DNA samples. The higher is the fetal fraction, the lower can coverage be.
2. The acceptable number of false positives and false negatives. As low coverage increases prediction variance, the z-score difference between true positives and true negatives decreases.

We analyzed separately samples (pooled both from training and validation dataset) in 8 coverage intervals (from 0.05 to 0.45 with interval size 0.05) and calculated the population standard deviation of model prediction for each interval. The results are shown on Figure S7.1.

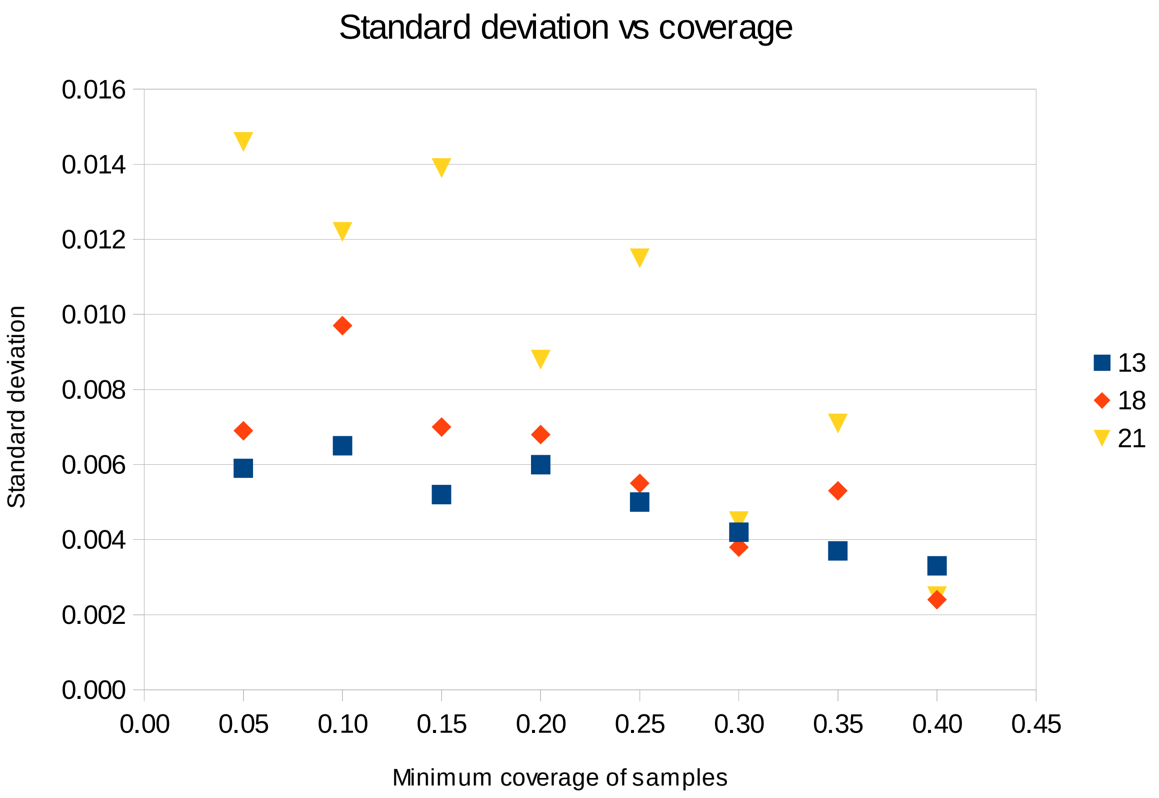

Figure S7.1
Standard deviations of prediction errors at different coverage intervals. X axis – minimum sequencing coverage in interval. Y axis – population standard deviation of prediction error. Two-component model (average + GC content) was used because the number of samples in each interval was too low for full model.

As one can see, at coverage 0.1 standard error of prediction is the range of 1%, depending on chromosome.

If the fetal fraction is, for example, 5% the true positive case has on average 2.5% higher coverage of affected chromosome. The average Z score of aneuploidy cases is thus 2.5.
At coverage 0.3 the standard error is in the range of 0.5% and we can expect the difference between control and aneuploidy cases to increases to 5 standard deviations.

Based on this analysis we expect that sequencing depth 0.1 is currently the minimum usable level where the aneuploidy cases can be detected with reasonable false negative and false positive rates.

One should still keep in mind, that the dispersion of experimental results may have other sources in addition to stochastic variance caused by low coverage. For example, DNA extraction, storage and sample preparation may induce sequence or methylation-dependent biases. Thus standardized methodology should be used across the whole sample set to minimize the variability.
Also, as is well-known in statistics, the dispersion estimates have much larger errors than the estimations of averages. Larger sample sets with more data points at different coverages are needed to more precisely estimate the true variance of per-chromosome coverages.
